# Inhibition of odontogenic differentiation of human dental pulp cells by dental resin monomers

**DOI:** 10.1186/s40824-015-0030-6

**Published:** 2015-04-10

**Authors:** Ji Hyun Kwon, Hee Chul Park, Tingting Zhu, Hyeong-Cheol Yang

**Affiliations:** Department of Dental Biomaterials Science and Dental Research Institute, School of Dentistry, Seoul National University, Chongro-ku, Seoul 110-749 Korea

**Keywords:** TEGDMA, HEMA, Hydrogen peroxide, Human dental pulp cell, Differentiation, Dental resin monomer, Odontogenic, MAP kinase

## Abstract

**Background:**

Dental resin monomers that are leached from the resin matrix due to incomplete polymerization can affect the viability and various functions of oral tissues and cells. In this study, the effects of triethylene glycol dimethacrylate (TEGDMA) and 2-hydroxyethyl methacrylate (HEMA) on odontogenic differentiation of human dental pulp cells (HDPCs) were examined. To mimic clinical situations, dental pulp cells were treated with resin monomers for 24 h prior to the analysis of alkaline phosphatase (ALP) activity and mRNA expression of genes related to pulp cell differentiation. To elucidate the underlying signaling pathways, regulation of mitogen-activated protein (MAP) kinases by resin monomers was also investigated.

**Results:**

The ALP activity of HDPCs was reduced by TEGDMA and HEMA at noncytotoxic concentrations. The mRNA expression of dentin sialophosphoprotein (DSPP), osteocalcin (OCN), and osteopontin (OPN) was also downregulated by resin monomers. However, DSPP expression was not affected by hydrogen peroxide (H_2_O_2_). Among the MAP kinases examined, ERK activation (ERK phosphorylation) was not affected by either resin monomers or H_2_O_2_, whereas JNK was phosphorylated by TEGDMA and HEMA. Phospho-p38 was upregulated by HEMA, while TEGDMA and H_2_O_2_ suppressed p38 phosphorylation.

**Conclusions:**

Exposure to TEGDMA and HEMA for a limited period suppresses differentiation of HDPCs via different signaling pathways.

## Background

Dental composite resins are predominantly used to restore damaged teeth. Restoration with resins is preferred for their ease of manipulation, lack of corrosion, and improved aesthetics. Composite resins contain viscous methacrylate monomers such as 2,2-bis[4-(2-hydroxy-3-methacrylyloxy-propoxy)phenyl] propane (bis-GMA) and urethane dimethacrylate (UDMA), in addition to hydrophilic monomers such as 2-hydroxyethyl methacrylate (HEMA) and triethylene glycol dimethacrylate (TEGDMA) [1-3]. Some resin monomers, especially HEMA and TEGDMA, are commonly identified in eluates of polymerized composite resins [4,5]. Resin monomers that are released due to incomplete polymerization of composite resins can penetrate into the pulp tissue and exert cytotoxic effects. The cytotoxic effects of HEMA and TEGDMA have been reported to depend on the exposure period and concentration [6,7]. Moreover, dental resin monomers have also been shown to impede odontogenic differentiation and the mineralization of dental pulp cells [8].

The root cause of resin monomer-induced side effects is oxidative stress, which is mediated by the formation of reactive oxygen species (ROS). For instance, TEGDMA-induced cell death and suppression of osteogenic differentiation have been shown to be blocked by the antioxidant N-acetylcysteine (NAC) [9]. Moreover, ROS can regulate the expression of redox-sensitive genes and activate signaling pathways for various cell functions, including proliferation and differentiation [10]. Among the ROS-mediated cell signaling pathways, the mitogen-activated protein (MAP) kinase pathway is known to be involved in cell survival and differentiation. In mammalian cells, MAP kinases are classified into different subfamilies, the ERK1/2 (extracellular signal-regulated kinase), JNK (c-Jun N terminal kinase), and p38 MAP kinases. The ERK pathway is part of the ras/raf/MEK pathway which is involved in cell proliferation and apoptosis [11,12]. The JNK and p38 pathways are related to stress-activated protein kinases (SAPKs), which play a role in oxidative stress, inflammation, and apoptosis [13-15]. Those proteins in MAP kinases are activated via phosphorylation. Although the functions of these pathways have been intensely investigated, the variation between and overlap of these pathways make it difficult to precisely determine which pathway is involved in a given set of circumstances.

Many studies have focused on the mechanisms of resin monomer-induced cytotoxicity, apoptosis, and cell death; however, pulp tissue function has been reported to be disrupted even at nontoxic concentrations of resin monomers. Previous *in vivo* studies have demonstrated that long-term exposure to nontoxic levels of resin monomers can induce inflammatory responses and exert adverse effects on pulp tissue function, which can suppress dentin regeneration [16,17]. Therefore, resin monomers can ultimately inhibit odontogenesis of human dental pulp cells (HDPCs). However, the mechanism underlying this inhibition has yet to be elucidated. In this study, we determined the effects of resin monomers on HDPC differentiation and investigated the mechanisms underlying these effects.

## Methods

### Chemicals and cell culture

All reagents were purchased from Sigma–Aldrich (St. Louis, MO, USA) unless otherwise noted. TEGDMA and HEMA were obtained from Aldrich Chemical Company (Deisenhofen, Germany). Anti-phospho-ERK, −JNK, and -p38 polyclonal antibodies, in addition to horseradish peroxidase-conjugated secondary antibodies, were purchased from Santa Cruz Biotechnology (Santa Cruz, CA, USA).

Under the approval of the Institutional Review Board of Seoul National University Dental Hospital, human dental pulp cells were extracted from incisors that had been removed from patients for orthodontic purposes. After swabbing teeth with 70% ethanol and phosphate-buffered saline (PBS) (pH 7.4), teeth were cut aseptically at the apex. Dental pulp was obtained from the pulp chamber and immersed in minimal essential medium (MEM) containing 20% fetal bovine serum (FBS) and antibiotic solution (100 U/mL of penicillin-G and 100 mg/mL of streptomycin). MEM and FBS were obtained from GIBCO-BRL (Carlsbad, CA, USA). The pulp was minced into several pieces and incubated at 37°C in a humidified atmosphere (5% CO_2_/95% air), with medium exchanges every 3 days. After 20 days, pulp cells were collected by treatment with trypsin solution and maintained in fresh medium. Cells were used in their fifth passage after confirming their alkaline phosphatase (ALP) activity in differentiation medium (MEM containing 50 mg/mL L-ascorbic acid, 10^−8^ M dexamethasone, and 2 mM β-glycerolphosphate) (9).

### Cytotoxicity test

HDPCs were incubated in 96-well plates until confluent and then treated with various concentrations of HEMA and TEGDMA. After treatment of resin monomers in growth medium which contained 10% FBS for 24 hrs, cell viability was measured using the WST-8 method as previously described [18]. Briefly, 10% WST-8 [2-(2-methoxy-4-nitrophenyl)-3-(4-nitrophenyl)-5-(2,4-disulfophenyl)-2H-tetrazolium, monosodium salt] (Dojindo Laboratories, Kumamoto, Japan) was added to culture medium without phenol red, and cells were incubated for 1 h. The absorbances were detected at a test wavelength of 450 nm and a reference wavelength of 600 nm using an automated microplate reader (Sunrise, TECAN, Salzburg, Austria). All experiments were performed in triplicate.

### ALP activity assay

The effects of resin monomers on HDPC ALP activity were evaluated using a 4-nitrophenyl phosphate-based colorimetric assay, as described previously [19]. HDPCs were exposed to 0.5 mM TEGDMA and 2 mM HEMA for 24 hrs in growth medium, and the cells were then incubated in growth medium or differentiation medium for 6 days before ALP activity assessment. Each well was washed with PBS, and then cells were incubated in a mixture of 140 μl of alkaline buffer solution (67 mM 4-nitrophenyl phosphate; Fluka, Buchs, Switzerland) and 10 μl of 1.5 mM MgCl_2_ solution for 30 min at 37°C. The reaction was stopped by the addition of 0.5 mM NaOH, and absorbances were measured at 405 nm. ALP activity is expressed as enzyme activity units per microgram of protein. Protein quantitation of each well was performed with a bicinchoninic acid (BCA) protein assay kit (iNtRON Biotechnology; Sungnam, Korea). To observe the effects of resin monomers after washout of the monomers from cell cultures, the treated cells were re-incubated in fresh medium for certain periods before initiation of differentiation.

### Gene expression analysis by real-time polymerase chain reaction

To investigate the effects of resin monomers on the differentiation of HDPCs, the mRNA levels of dentin sialophosphoprotein (DSPP), osteocalcin (OCN), and osteopontin (OPN) were measured by real-time polymerase chain reaction (RT-PCR). After treatment with resin monomers for 24 hrs, cells were washed and maintained in differentiation medium for 12 days. HDPCs treated with 0.2 mM H_2_O_2_ were used as a control for oxidative stress. Total RNA was obtained with the WelPrep Total RNA Isolation Reagent (Welgene Inc, Daegu, Korea), and cDNA was prepared with a Power cDNA Synthesis kit (iNtRON Biotechnology). Real-time PCR was performed in a mixture of 10 μl SYBR Premix Ex Taq (Takara Bio, Otsu, Japan), 0.4 μl ROX Reference Dye II (Takara Bio), cDNA, and primers on an ABI PRISM 7500 Sequence Detection System Thermal Cycler (Applied Biosystems, Foster City, CA, USA). The following primers were used: DSPP, forward 5′-GCATTCAGGGACAAGTAAGCA-3′, reverse 5′-CTTGGACAACAGCGACATCCT-3′; OCN, forward 5′-GTGACGAGTTGGCTGACC-3′, reverse 5′-CAAGGGGAAGAGGAAAGAAGG-3′; OPN, forward 5′-CAGACGAGGACATCACCTCA-3′, reverse 5′-TGGCTGTGGGTTTCAGCA-3′; glyceraldehyde-3-phosphate dehydrogenase (GAPDH), forward 5′-GTCGGAGTCAACGGATTTGG-3′, reverse 5′-GGGTGGAATCA ATTGGAACATG-3′. The PCR thermocycling conditions were: 95°C for 30 sec, followed by 40 cycles of denaturation at 95°C for 15 sec and annealing at 60°C for 30 sec. DSPP, OCN, and OPN expression levels were calculated based on their threshold cycle (C_T_) values and are expressed as relative mRNA expression ratios normalized to a reference gene (GAPDH).

### Protein expression analysis by western blotting

The expression of phospho-ERK, JNK, and p38 in resin-treated cells was observed by western blotting. HDPCs were incubated in 6-well plates until confluent, and then cells were washed and treated with resin monomers for 1, 3, and 6 hrs. HDPCs treated with 0.2 mM H_2_O_2_ were used as a positive control. Proteins were extracted in cold NP-40 lysis buffer [50 mM Tris–HCl (pH 7.6); 150 mM NaCl; 10% glycerol; 1% NP-40; 1 mM phenylmethylsulfonyl fluoride; and 1 μg/mL each of leupeptin, aprotinin, and pepstatin] for 15 min at 4°C and lysates were then spun by centrifugation at 14,000 × *g* for 10 min at 4°C. The total protein concentrations of the lysates were measured using a Pro-Measure kit (iNtRON Biotechnology, Seoul, Korea). Equal amounts of protein (30 μg) were subjected to SDS-polyacrylamide gel electrophoresis on 10% gels and then transferred to polyvinylidene difluoride (PVDF) transfer membranes (Hybond-P; Amersham Biosciences, Bucks, England). After blocking [6% (w/v) dried low-fat milk and 0.1% (v/v) Tween 20 in PBS (PBST)], the blots were incubated with anti-phospho-ERK, −JNK, and -p38 polyclonal antibodies in PBST for 1 hr followed by two washes (15 min each) in PBST. The blots were then probed with goat anti-rabbit secondary antibodies conjugated to horseradish peroxidase. Immunoreactive bands were visualized using a chemiluminescence kit (WEST-ZOL plus Western Blot Detection System; iNtRON Biotechnology, Seoul, Korea). Chemiluminescence was detected with a MicroChemi Bio-image analyzer (DNR, Jerusalem, Israel).

### Statistical analysis

Data are expressed as means ± SDs of three or more experiments. The significance of differences between control and treated groups was analyzed using the paired Student’s *t*-test.

## Results

### Effects of resin monomers on HDPC viability

**Figure 1:**
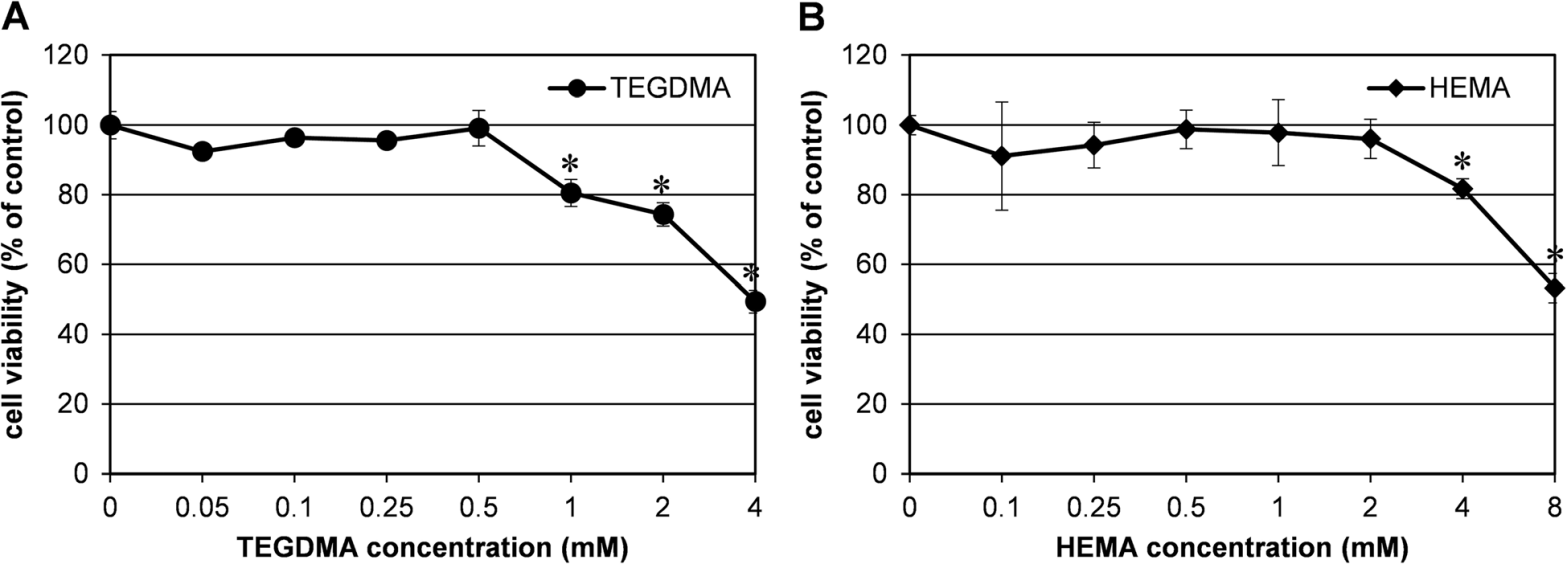
**Effects of TEGDMA (A) and HEMA (B) on HDPC viability.** Cells were exposed to TEGDMA and HEMA for 24 hrs and then cell viability was evaluated. Each error bar represents the standard deviation of three independent experiments. * indicates significant (*p* < 0.05) differences between the control and test groups.

To observe the effects of the different resin monomers on HDPC viability, pulp cells were treated with resin monomers at various concentrations for 24 hrs (Figure 1). HEMA and TEGDMA exhibited cytotoxicity at concentrations above 4 and 1 mM, respectively. HEMA reduced cell viability by 81.7% at 8 mM. In the case of TEGDMA treatment, cell viability was reduced by 49.4 and 80.5% at 1 and 4 mM, respectively.

### Effects of resin monomers on ALP activity of HDPCs

**Figure 2:**
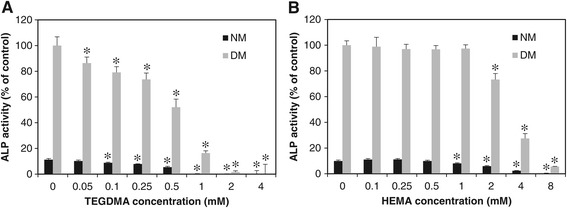
**Effects of TEGDMA (A) and HEMA (B) on HDPC ALP activity.** Cells were exposed to 0.5 mM TEGDMA and 2 mM HEMA for 24 hrs. Before assaying ALP activity, cells were incubated in growth medium (NM) or differentiation medium (DM) for 6 days. ALP activity (units/mg protein) is expressed as% of the control. Each error bar represents the standard deviation of three independent experiments. * indicates significant (p < 0.05) differences between the control and test groups.

**Figure 3:**
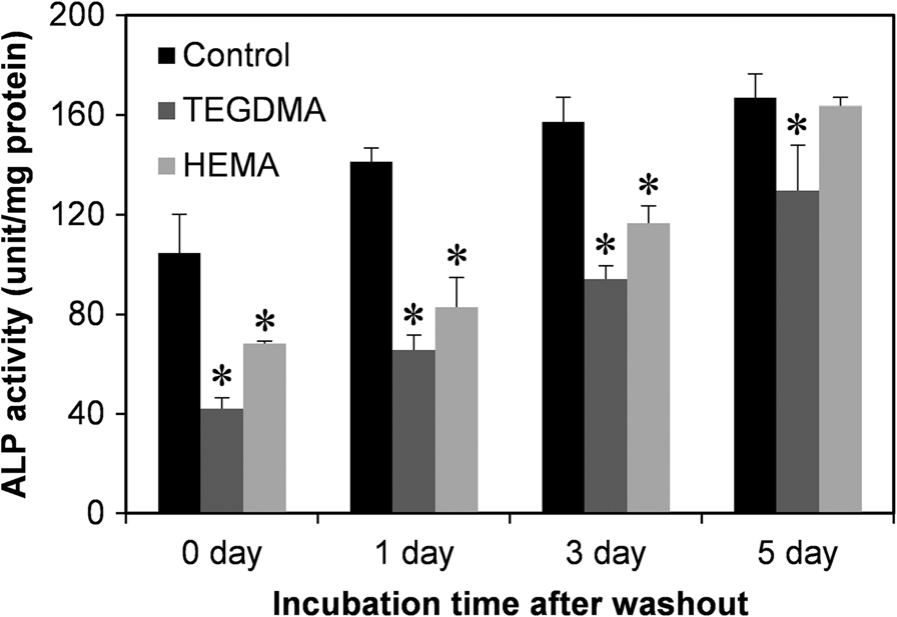
**Recovery of ALP activity by washout of resin monomers.** HDPCs were treated with 0.5 mM TEGDMA and 2 mM HEMA for 24 hrs, and resin monomers were washed out for certain periods by incubating in fresh medium. ALP activity was measured after the activation of differentiation in differentiation medium for 6 days. * indicates significant (p < 0.05) differences between the control and test groups.

Next, the effects of HEMA and TEGDMA on the ALP activity of HDPCs were investigated. Previous studies primarily investigated the changes of ALP activity in dental pulp cells that were continuously exposed to resin monomers. In this study, however, HDPCs were exposed to resin monomers for only 24 hrs in normal medium and then incubated in differentiation medium for 6 days without resin monomers before ALP measurement. As shown in Figure 2A and B, exposure to HEMA and TEGDMA resulted in concentration-dependent decreases of ALP activity in HDPCs. HEMA began to inhibit ALP activity at concentrations higher than 2 mM, and ALP activity was completely abolished at 8 mM. However, the range of concentrations that inhibited ALP activity corresponded to the range of concentrations that affected cell viability, suggesting that the effect was not specific. In contrast, ALP activity of HDPCs was highly susceptible to TEGDMA, even at concentrations that did not affect cell viability. For example, treatment with 0.05 mM HEMA resulted in a significant decrease in ALP activity, whereas cell viability was not altered even at concentrations up to 0.5 mM. This result indicates that treatment with HEMA for 24 hrs is enough to affect cell differentiation. We also investigated the durations of the effects of resin monomers in HDPCs. Cells that had been treated with resin monomers for 24 hrs were then incubated in fresh medium (monomer washout) for the indicated times and finally incubated in differentiation medium for 6 days to induce differentiation. The concentration of TEGDMA and HEMA was 0.5 mM and 2 mM respectively, the highest non-cytotoxic concentrations. As shown in Figure 3, HDPC ALP activity recovered as the washout time increased. Specifically, the ALP activity of cells that were treated with 2 mM HEMA returned to the level of untreated cells when the cells were reincubated for 5 days in fresh medium before induction of differentiation. TEGDMA-treated cells also recovered more ALP activity with longer washout periods.

### Effects of resin monomers on the mRNA expression of DSPP, OCN, and OPN

**Figure 4:**
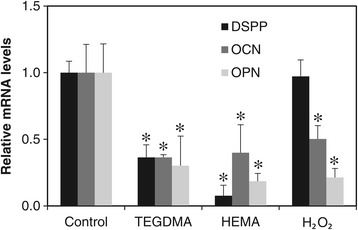
**Effects of TEGDMA, HEMA, and H**_**2**_ **O**_**2**_ **on the mRNA expression levels of DSPP, OCN, and OPN.** Cells were treated with 0.5 mM TEGDMA, 2 mM HEMA, or 0.2 mM H_2_O_2_ for 24 hrs. Each error bar represents the standard deviation of three independent experiments. * indicates significant (p < 0.05) differences between the control and test groups.

Next, the effects of resin monomers on HDPC differentiation were investigated on the molecular level. Cells were treated with HEMA, TEGDMA, and H_2_O_2_ for 24 hrs and then incubated in differentiation medium lacking resin monomers and H_2_O_2_ for 12 days prior to the quantitation of DSPP, OCN, and OPN mRNA expression by real-time PCR. As shown in Figure 4, TEGDMA and HEMA significantly reduced the mRNA expression of DSPP, OCN, and OPN. The expression of DSPP, an odontogenic differentiation-specific gene, was downregulated the most in HEMA-treated cells; in contrast, H_2_O_2_ did not affect DSPP expression. In H_2_O_2_–treated cells, OPN expression was more severely affected than OCN expression. The effects of TEGDMA were similar among the three genes.

### Effects of resin monomers on MAP kinase protein expression

**Figure 5:**
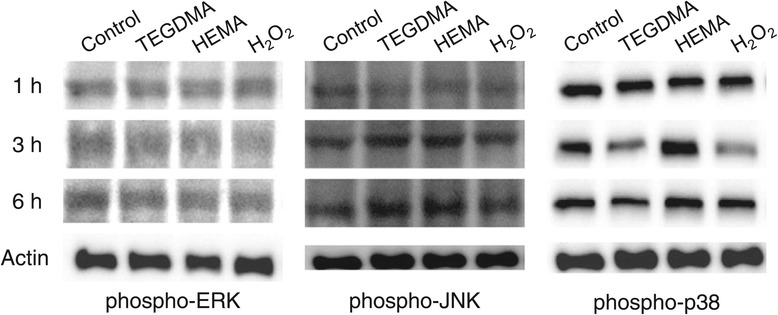
**Effects of TEGDMA, HEMA, and H**_**2**_ **O**_**2**_ **on the expression of MAP kinase proteins (ERK, JNK, and p38).** Cells were treated with 0.5 mM TEGDMA, 2 mM HEMA, and 0.2 mM H_2_O_2_ for 1, 3, and 6 hrs. A representative image from three independent experiments is shown.

To investigate the mechanism underlying resin monomer-induced inhibition of odontogenesis, the expression of various MAP kinases (phospho-ERK, JNK, and p38) in HDPCs was observed. HDPCs were treated with TEGDMA, HEMA, and H_2_O_2_ for 1, 3, and 6 hrs. As shown in Figure 5, expression of phospho-ERK was not altered by treatment with resin monomers or with H_2_O_2_. However, JNK and p38 exhibited different responses to these agents. Both TEGDMA and HEMA activated JNK, as demonstrated by the increased levels of phospho-JNK. At 3 and 6 hr, the amounts of phospho-JNK were increased, whereas H_2_O_2_ did not affect JNK activation. Regarding p38 activation, the level of phospho-p38 was slightly elevated in HEMA-treated cells at 3 hr. However, this effect was attenuated at 6 hr. In contrast to HEMA, both TEGDMA and H_2_O_2_ suppressed p38 activation at 3 hr.

## Discussion

Since the apex of a tooth is generally open, chemicals in the pulp cavity can diffuse outside the tooth. Thus, hydrophilic dental resin monomers of the resin matrix are not trapped in the pulp cavity but are removed from the tooth by diffusion or transport via blood vessels. Considering the open properties of the pulp cavity, the effects of resin monomers were investigated with HDPCs that were treated with resin monomers for limited periods. As shown in Figure 1, TEGDMA was more cytotoxic than HEMA, which is consistent with previous studies [20,21]. This difference in cytotoxicity has been explained by their contrasting lipophilicities in culture medium, due to their distinct molecular structures [22]. TEGDMA and HEMA were noncytotoxic at 0.5 and 2 mM, respectively, concentrations at which the differentiation of dental pulp cells was significantly inhibited. Therefore, the inhibition of differentiation observed in the present study was not a nonspecific effect due to resin monomer cytotoxicity. We treated cells with resin monomers for only 24 hrs in the present study. This duration was chosen to mimic the clinical situation, since most monomers have been shown to leach from the resin matrix during the first day after polymerization [23]. Therefore, the results in Figure 2 indicate that TEGDMA and HEMA in the pulp cavity can potentially interfere with odontogenic differentiation of pulp cells, even if the contact between resin monomers and cells is only temporary. We also determined whether the anti-differentiation effect of these monomers was reversible or not. To this end, the monomers were washed out of monomer-treated cells for different periods and the cells were then allowed to differentiate. As shown in Figure 3, increasing the washout time also increased the recovery of ALP activity, demonstrating that the effect of resin monomers is attenuated by prolonged washout periods and that the monomer-induced damage is reversible. This result also suggests that the ability of dental pulp cells to differentiate is not permanently damaged unless their viability is affected. Since this hypothesis is based only on our ALP activity results, future studies with other differentiation markers are needed.

Treatment of cells with resin monomers for 24 hrs downregulated the expression of multiple differentiation-related genes and also reduced ALP activity. Interestingly, TEGDMA, HEMA, and H_2_O_2_ had different effects on gene expression and ALP activity. DSPP, a representative marker of odontogenic differentiation, was drastically downregulated by HEMA, while H_2_O_2_ did not affect DSPP mRNA expression. Interestingly, a previous study found that HEMA-mediated inhibition of cell differentiation was accompanied by oxidative stress [9]. However, the strikingly different effects of HEMA and H_2_O_2_ on DSPP mRNA expression suggest that the types of reactive oxygen species (ROS) produced and the oxidative stress signaling pathways activated in HEMA-treated cells are not identical to those in H_2_O_2_-treated cells. Consistent with the idea that distinct mechanisms are involved, TEGDMA treatment also resulted in a different pattern of mRNA expression.

Since MAP kinases are known to play an important role in signaling transduction during oxidative stress, resin monomer-treated cells were predicted to have altered MAP kinase expression. Among the three MAP kinases shown in Figure 5, the expression levels of both JNK and p38 were altered by TEGDMA and HEMA. Specifically, the level of phospho-JNK was increased by treatment with both TEGDMA and HEMA. On the other hand, TEGDMA downregulated phospho-p38 expression, whereas HEMA upregulated phospho-p38 expression. H_2_O_2_ affected p38 activation, but not JNK activation. Downregulation of p38 by both TEGDMA and H_2_O_2_ has previously been demonstrated [24]. These results indicate that TEGDMA, HEMA, and H_2_O_2_ activate different oxidative stress signaling pathways.

## Conclusion

Noncytotoxic concentrations of HEMA and TEGDMA reduced ALP activity and downregulated the mRNA expression of multiple odontogenic genes (DSPP, OCN, and OPN) in HDPCs. The p38 signaling pathway appears to be at least partially involved in TEGDMA-mediated suppression of ALP activity; however, additional studies are needed to fully elucidate the mechanisms of these processes.
